# Practical Pathological Assessment of Residual Cancer Burden (RCB) After Neoadjuvant Therapy in Human Epidermal Growth Factor Receptor 2 (HER2)-Positive Invasive Breast Carcinoma: A Case Report

**DOI:** 10.7759/cureus.117596

**Published:** 2026-10-07

**Authors:** Gunvanti Rathod, Chandramouli R, Immanuel Pradeep, Ajay Kumar Kondeti

**Affiliations:** 1 Department of Pathology and Laboratory Medicine, All India Institute of Medical Sciences, Bibinagar, Bibinagar, IND; 2 Department of Radiation Oncology, All India Institute of Medical Sciences, Bibinagar, Bibinagar, IND

**Keywords:** adjuvant chemotherapy, breast carcinoma, her2-positive, neoadjuvant chemotherapy, residual cancer burden (rcb) index

## Abstract

Neoadjuvant therapy is routinely used in human epidermal growth factor receptor 2 (HER2)-positive breast carcinoma and permits the assessment of treatment response in the surgical specimen. Although pathological complete response (pCR) is an important endpoint, it is binary and does not quantify the extent of residual disease in patients with non-pCR. The Residual Cancer Burden (RCB) index provides a standardized quantitative assessment incorporating the dimensions of the treated tumor bed, residual cancer cellularity, in situ component, and nodal involvement. We report a 48-year-old woman with HER2-positive, estrogen receptor/progesterone receptor (ER/PR)-negative invasive breast carcinoma who received six cycles of neoadjuvant TCH (docetaxel, carboplatin, and trastuzumab) followed by modified radical mastectomy. Gross examination showed no residual mass but identified a fibrotic tumor bed measuring 7 × 5 × 5 cm. Microscopy demonstrated a single residual invasive focus measuring 0.4 × 0.4 cm with 10% overall cancer cellularity, 1% in situ component, and no metastatic tumor in 13 axillary lymph nodes. The RCB index was 1.891 (RCB-II), with final staging of ypT1aN0, and adjuvant ado-trastuzumab emtansine (T-DM1) was initiated. This case is not novel in RCB or HER2-directed therapy, but it demonstrates the importance of meticulous tumor-bed evaluation after neoadjuvant chemotherapy. Treatment-related fibrosis and inflammatory change may obscure scattered residual carcinoma, and a small residual invasive focus may coexist with a substantially larger treated tumor bed. Accurate RCB assessment therefore requires correlation of gross findings, microscopy, nodal evaluation, and pre-treatment disease location. In this setting, RCB complements pCR by providing a reproducible description of residual disease that can support multidisciplinary risk assessment and management.

## Introduction

Breast cancer is the most commonly diagnosed malignancy in women worldwide and is biologically heterogeneous. Human epidermal growth factor receptor 2 (HER2)-positive breast carcinoma, comprising 15-20% of cases, is characterized by aggressive behavior and high proliferation. The advent of HER2-targeted therapy, particularly trastuzumab, has significantly improved outcomes [1].

Neoadjuvant systemic therapy is routinely used in such tumors, enabling tumor downstaging and in vivo response assessment [2]. Traditionally, response is evaluated as pathological complete response (pCR) or not, but this binary measure fails to capture residual disease heterogeneity [3]. 

The Residual Cancer Burden (RCB) index offers a standardized quantitative assessment, incorporating four tumor parameters, namely, the largest two dimensions of the residual primary tumor bed, overall cancer cellularity, the proportion of cancer represented by in situ disease, and two nodal parameters (the number of positive lymph nodes and the size of the largest nodal metastasis when present), classifying responses into four prognostic categories [4]. These categories correlate with long-term outcomes and provide improved prognostic stratification [5].

We present this case as a pathology-focused illustration of the practical application of RCB assessment after neoadjuvant TCH (docetaxel, carboplatin, and trastuzumab) in HER2-positive breast carcinoma. The emphasis is on the post-treatment specimen: recognition of the treated tumor bed, identification of residual invasive carcinoma within therapy-related changes, appropriate interpretation of tumor-bed dimensions and cellularity, proportion of ductal carcinoma in situ (DCIS), and integration of nodal findings for RCB calculation. These steps are clinically relevant because the gross absence of a tumor mass does not establish pCR and because residual disease may be sparse or discontinuous within a fibrotic bed [6].

## Case presentation

Clinical presentation 

A 48-year-old postmenopausal woman presented with a right breast lump of one year's duration, with no significant family history and a background of hypothyroidism on regular treatment. Clinical staging was cT2N0M0 (Stage IIA). A mammogram revealed a well-defined radiodense lesion in the outer quadrant of the right breast measuring 2.3 × 2.1 × 2.5 cm (Breast Imaging Reporting and Data System (BI-RADS) IVb), with no contralateral lesion. Bilateral axillae showed small benign-appearing lymph nodes (Figure 1).

**Figure 1:**
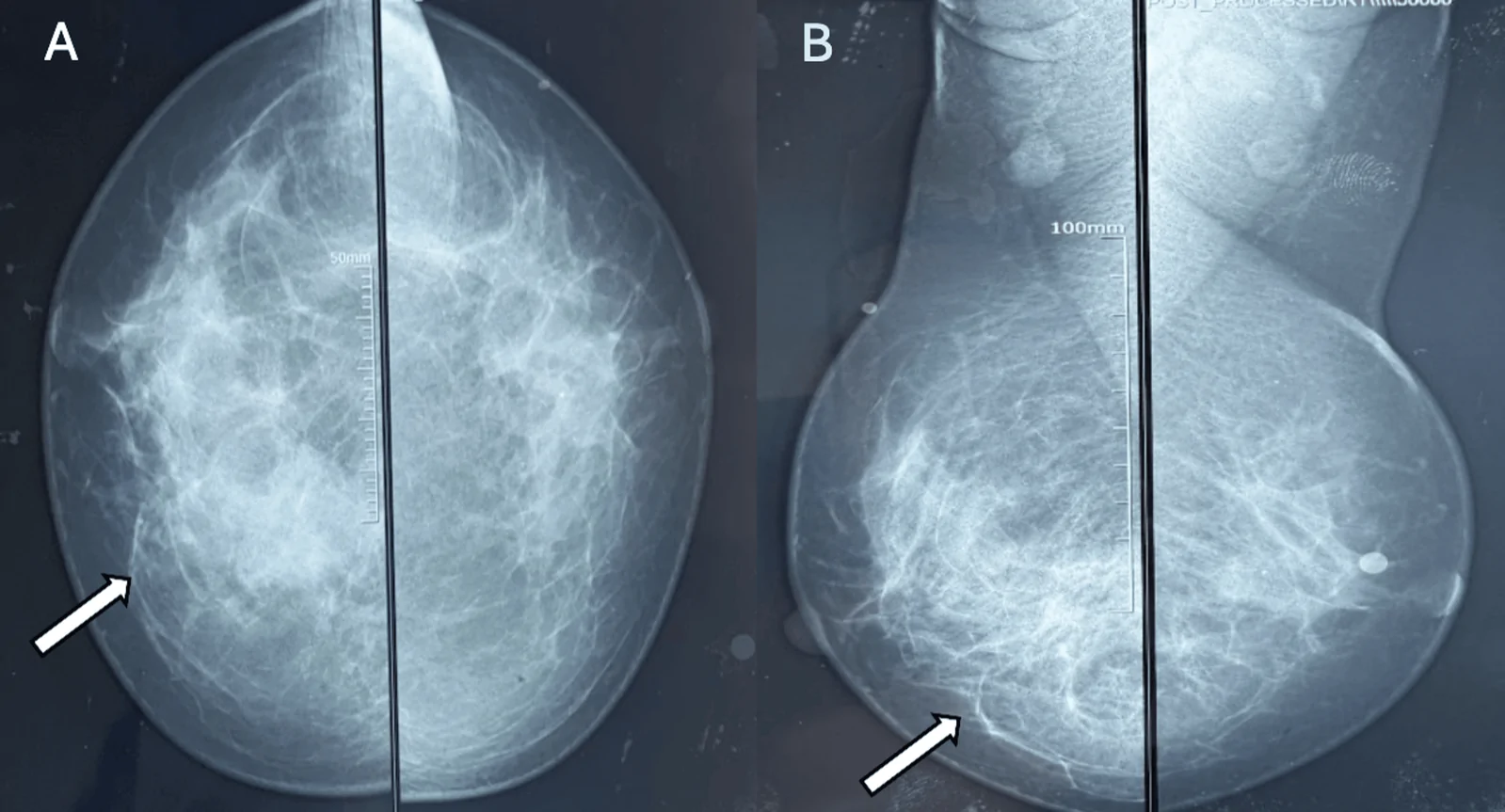
Mammogram Mammogram revealed a well-defined radiodense lesion (indicated by the white arrows) in the outer quadrant of the right breast measuring 2.3 × 2.1 × 2.5 cm (BI-RADS IVb): (A) CC view and (B) MLO view. BI-RADS: Breast Imaging Reporting and Data System; CC: craniocaudal; MLO: mediolateral oblique

Contrast-enhanced CT of the chest demonstrated a 2.6 × 2.3 cm heterogeneously enhancing lesion without mediastinal lymphadenopathy or distant metastases (Figure 2).

**Figure 2:**
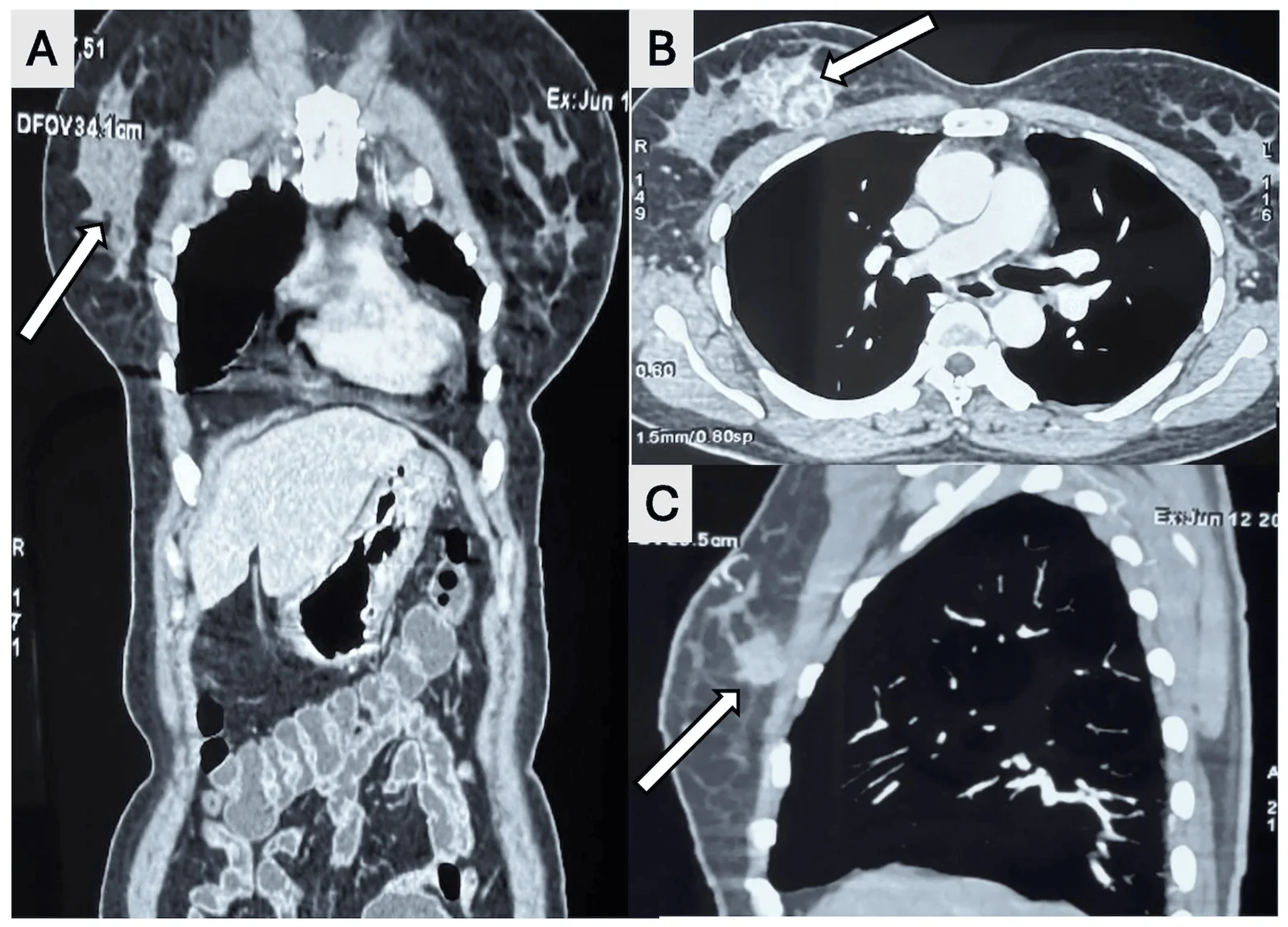
Chest CT scan CT scan of the chest showing a heterogeneously enhancing lesion (indicated by the white arrows) in the right breast: (A) coronal view, (B) axial view, and (C) sagittal view.

Core biopsy confirmed invasive breast carcinoma, no special type, Nottingham grade 2, with lymphovascular invasion. Immunohistochemistry showed HER2 positivity (3+), estrogen receptor/progesterone receptor (ER/PR) negativity (Allred 0/8), and a Ki-67 index of 35-40% (Figure 3).

**Figure 3:**
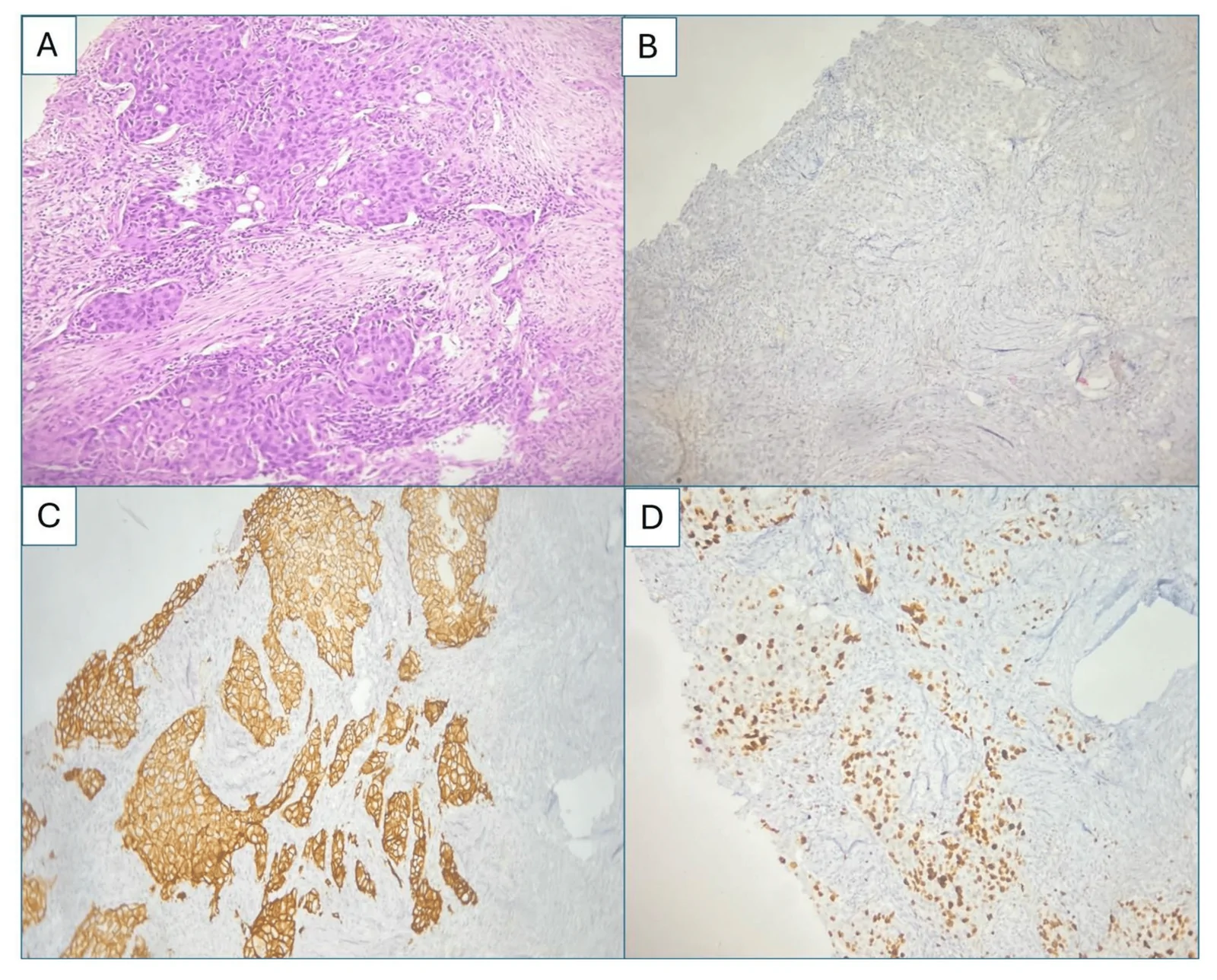
Breast tumor core biopsy Breast tumor core biopsy findings: (A) invasive breast carcinoma, NST, grade 2 (H&E stain, ×100), (B) IHC for estrogen receptor-negative (Allred score 0/8), (C) IHC for Her2neu-positive (score 3), and (D) IHC for Ki-67 labelling index is approximately 35-40%. NST: no special type; H&E: hematoxylin and eosin; IHC: immunohistochemistry

Based on the multidisciplinary team decision, neoadjuvant systemic therapy was advised for this HER2-positive, hormone receptor-negative carcinoma [7]. She received six cycles of the TCH regimen over four months with good tolerance and clinical response.

Subsequently, she underwent a right modified radical mastectomy. Gross examination showed no discrete residual tumor mass; however, a fibrotic tumor bed measuring 7 × 5 × 5 cm was identified. All margins were negative, with the closest (deep) margin at 5 mm. Thirteen axillary lymph nodes were dissected, all negative for malignancy. The discrepancy between the absence of a gross residual mass and the presence of a definable treated tumor bed was important for subsequent microscopic evaluation and RCB assessment.

Microscopic examination of the specimen showed invasive carcinoma of no special type (ductal), histologic grade 2, as per Nottingham histologic score 7 [8]. A single residual invasive focus measuring 0.4 × 0.4 cm was identified within the treated tumor bed, with 10% overall cancer cellularity and 1% in situ component. DCIS was present, of solid type, high nuclear grade, with comedo necrosis. No lymphovascular invasion was identified in the post-therapy specimen. A probable or definite response to pre-surgical therapy was noted in the invasive carcinoma. Margins were negative for invasive tumor and DCIS, and no tumor was identified in the 13 examined lymph nodes (Figure 4). Thus, although the residual invasive component was small, viable carcinoma remained within a substantially larger therapy-related fibrotic bed.

**Figure 4:**
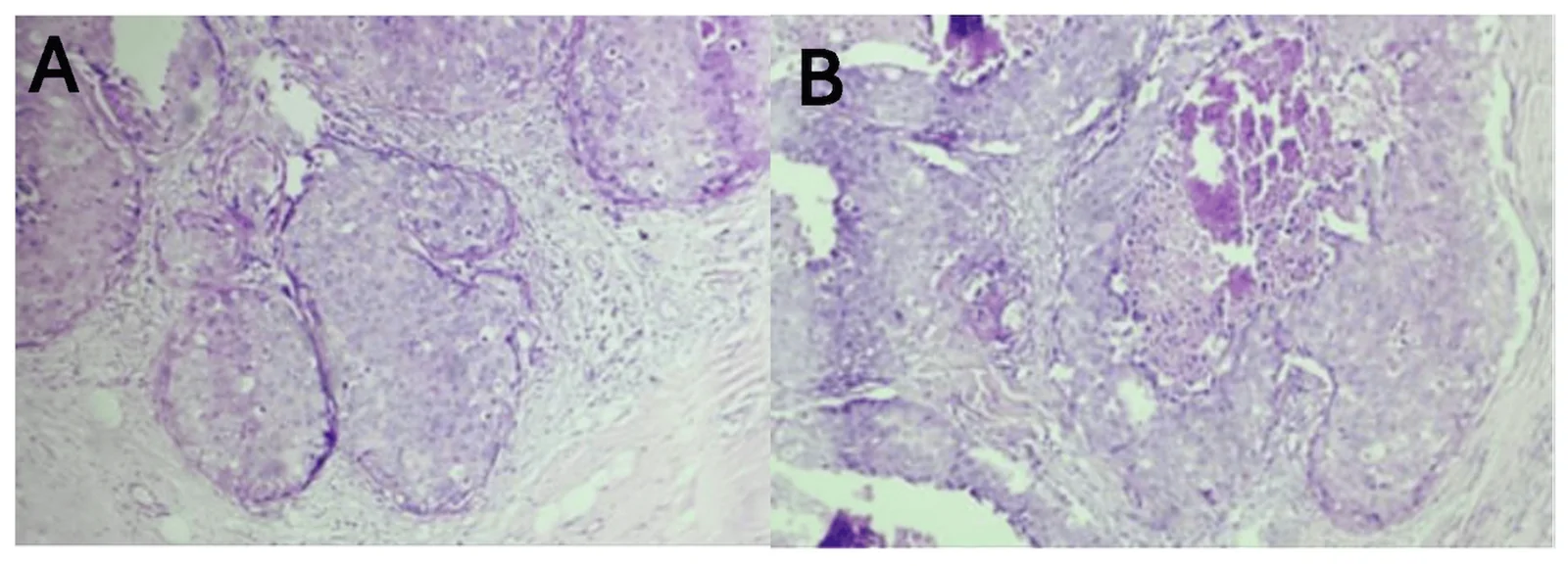
Mastectomy specimen microscopic findings Microscopic examination of the post-neoadjuvant systemic therapy mastectomy specimen. (A) An H&E-stained section shows infiltrating malignant epithelial cells arranged in cords and nests within a desmoplastic stromal background (10×). (B) An H&E-stained section shows malignant epithelial cells with the presence of necrosis. Tumor cells exhibit enlarged pleomorphic nuclei and moderate eosinophilic cytoplasm (10×). H&E: hematoxylin and eosin

The RCB index was calculated using the UT MD Anderson Residual Cancer Burden calculator [4]. The documented parameters were a primary tumor bed measuring 70 × 50 mm for RCB calculation, a residual invasive tumor measuring 0.4 × 0.4 cm, an overall cancer cellularity of 10%, an in situ fraction of 1%, and zero positive lymph nodes. The calculated RCB index was 1.891, corresponding to RCB Class II (moderate residual disease). Final pathologic staging was ypT1aN0 as per the American Joint Committee on Cancer (AJCC) 8th edition [9]. Importantly, the RCB classification was based on the integrated residual disease measurements rather than on the size of the single residual invasive focus alone.

In view of residual invasive HER2-positive disease after neoadjuvant therapy, adjuvant ado-trastuzumab emtansine (T-DM1) was initiated. The therapeutic decision is mentioned to demonstrate the clinical consequence of identifying residual disease, rather than to suggest that the treatment itself is novel.

## Discussion

Neoadjuvant therapy has become an established strategy for HER2-positive and other biologically aggressive breast cancers. Its value extends beyond tumor downstaging because the resection specimen provides an opportunity to assess the degree of treatment response. pCR is prognostically important, but it is a binary endpoint and does not distinguish among the different amounts and patterns of residual disease in patients who do not achieve pCR [2,3]. 

The RCB system was developed as a standardized quantitative approach to post-neoadjuvant assessment. It incorporates the largest two dimensions of the residual primary tumor bed, overall cancer cellularity, the proportion of cancer represented by in situ disease, the number of positive lymph nodes, and the size of the largest nodal metastasis when present [4]. The resulting continuous RCB index is assigned to RCB-0, RCB-I, RCB-II, or RCB-III categories. RCB therefore complements, rather than replaces, conventional pathologic staging and assessment of treatment effect [4,5].

Post-neoadjuvant specimen evaluation: practical pathological considerations

The post-neoadjuvant breast specimen can be deceptively difficult to evaluate. Treatment may produce fibrosis, edema, necrosis, inflammation, histiocytic aggregates, and other stromal changes that obscure or separate residual malignant cells. With a marked response, residual carcinoma may be sparse or occur as multiple small foci distributed through a larger fibrotic tumor bed. Therefore, the radiologic site and pre-treatment tumor location should be correlated with the gross specimen, and the treated tumor bed should be identified and adequately sampled. International recommendations emphasize radiologic-pathologic correlation, identification and mapping of the treated tumor bed, adequate sampling, and standardized reporting of residual invasive disease, DCIS, treatment effect, nodal status, and RCB [6,10].

Gross absence of a residual mass should not be equated with pCR. In the present case, no gross tumor was identified, yet a 7 × 5 × 5 cm fibrotic tumor bed contained a 0.4 × 0.4 cm residual invasive focus. This illustrates why the treated tumor bed, rather than only a grossly visible residual mass, must guide microscopic evaluation. The pathologist should document the tumor-bed dimensions, describe the residual invasive component and its cellularity, distinguish invasive carcinoma from residual DCIS, and evaluate the regional lymph nodes carefully. The largest residual invasive focus determines the post-treatment ypT category, whereas RCB integrates tumor-bed area and cellularity with the nodal component [4,6,10].

Several practical pitfalls deserve emphasis. First, therapy-related fibrosis surrounding residual carcinoma should not be measured as invasive tumor for ypT staging. Second, the extent of the treated tumor bed and the size of the largest residual invasive focus are not interchangeable variables. Third, residual DCIS contributes to the RCB calculation through the in situ fraction but is not equivalent to residual invasive disease. Fourth, scattered residual carcinoma may be missed if sampling is limited to an apparently scarred or grossly unremarkable area. Fifth, nodal treatment response must be incorporated because residual nodal disease contributes to RCB even when the breast shows marked regression. Finally, treatment may alter morphology and, in some cases, biomarker expression; correlation with the pre-treatment biopsy and appropriate repeat biomarker assessment should be considered according to clinical and laboratory protocols [6,10].

These considerations give the present case its practical relevance. The apparent discrepancy between a very small residual invasive focus and the RCB-II category can be understood because RCB is not a tumor-size-only score. It integrates the extent of the treated tumor bed, residual cellularity, in situ component, and nodal status. Thus, reporting only "0.4 cm residual carcinoma" would underrepresent the information conveyed by the complete post-treatment assessment. Documenting the variables required for RCB calculation makes the report more reproducible and facilitates communication with the multidisciplinary team [4,10].

RCB should also be interpreted alongside other response-assessment systems. Miller-Payne and Sataloff provide useful qualitative descriptions of tumor regression, whereas RCB provides a quantitative estimate of residual disease [11,12]. Increasing RCB class has been associated with progressively less favorable outcomes in validation studies, including HER2-positive disease [3,5]. In clinical practice, however, the RCB score should be interpreted in the context of tumor biology, pre-treatment stage, nodal status, treatment regimen, and the final pathologic findings rather than used in isolation.

In the present case, good treatment response was evident, but the microscopic finding of residual invasive carcinoma prevented classification as pCR. The RCB index of 1.891 placed the patient in RCB-II despite only a 0.4 cm residual invasive focus and no nodal metastasis. This apparently counterintuitive result illustrates the central practical point of RCB: residual disease is quantified using several pathologic variables rather than the largest invasive focus alone.

The therapeutic implication in this patient is consistent with established evidence rather than representing a novel treatment observation. In HER2-positive breast carcinoma with residual invasive disease after neoadjuvant therapy, the KATHERINE trial demonstrated improved outcomes with adjuvant T-DM1 compared with trastuzumab [13]. In the present case, identification of residual invasive disease and its RCB classification therefore contributes to multidisciplinary treatment planning.

The principal educational contribution of this case is the close link between meticulous post-neoadjuvant chemotherapy pathology and clinically meaningful residual-disease stratification. It demonstrates that grossly absent tumor does not necessarily indicate pCR, that a small residual invasive focus may be embedded within a much larger fibrotic tumor bed, and that RCB-II cannot be inferred from residual tumor size alone. As a single case report, it cannot establish the superiority of RCB over other response-assessment systems or generate new therapeutic evidence; its value lies in illustrating a reproducible, pathology-centered approach to a common post-neoadjuvant diagnostic problem.

Overall, the case supports a practical reporting approach in which the post-neoadjuvant chemotherapy specimen is assessed systematically for the treated tumor bed, residual invasive carcinoma, in situ disease, treatment-related changes, margins, and regional lymph nodes, followed by RCB calculation when appropriate. This approach helps translate routine microscopic observations into a standardized residual-disease measure that can be communicated effectively to the multidisciplinary team.

Follow-up plan

Based on the National Comprehensive Cancer Network (NCCN) guidelines, escalated therapy with adjuvant T-DM1 was initiated at three-week intervals for 14 cycles. No post-mastectomy radiotherapy (PMRT) was indicated. No hormonal therapy was indicated in view of the ER/PR-negative status.

## Conclusions

The RCB index provides a standardized and clinically meaningful assessment of residual disease following neoadjuvant chemotherapy. This case highlights the importance of meticulous tumor-bed evaluation and adequate pathological sampling, particularly when minimal residual invasive disease is present. Despite ypT1aN0 staging, RCB-II classification demonstrated moderate residual disease and supplements the information beyond pCR and conventional staging, supporting its integration into routine post-neoadjuvant pathological reporting and multidisciplinary treatment planning.
